# Inflammation-related genes and immune infiltration landscape identified in kainite-induced temporal lobe epilepsy based on integrated bioinformatics analysis

**DOI:** 10.3389/fnins.2022.996368

**Published:** 2022-10-27

**Authors:** Li Wang, Chunmei Duan, Ruodan Wang, Lifa Chen, Yue Wang

**Affiliations:** Department of Neurology, Xinqiao Hospital and The Second Affiliated Hospital, Army Medical University, Chongqing, China

**Keywords:** temporal lobe epilepsy, PPI, WGCNA, immunocyte infiltration, neuroinflammatory, kainite

## Abstract

**Background:**

Temporal lobe epilepsy (TLE) is a common brain disease. However, the pathogenesis of TLE and its relationship with immune infiltration remains unclear. We attempted to identify inflammation-related genes (IRGs) and the immune cell infiltration pattern involved in the pathological process of TLE *via* bioinformatics analysis.

**Materials and methods:**

The GSE88992 dataset was downloaded from the Gene Expression Omnibus (GEO) database to perform differentially expressed genes screening and weighted gene co-expression network analysis (WGCNA). Subsequently, the functional enrichment analysis was performed to explore the biological function of the differentially expressed IRGs (DEIRGs). The hub genes were further identified by the CytoHubba algorithm and validated by an external dataset (GSE60772). Furthermore, the CIBERSORT algorithm was applied to assess the differential immune cell infiltration between control and TLE groups. Finally, we used the DGIbd database to screen the candidate drugs for TLE.

**Results:**

34 DEIRGs (33 up-regulated and 1 down-regulated gene) were identified, and they were significantly enriched in inflammation- and immune-related pathways. Subsequently, 4 hub DEIRGs (Ptgs2, Jun, Icam1, Il6) were further identified. Immune cell infiltration analysis revealed that T cells CD4 memory resting, NK cells activated, Monocytes and Dendritic cells activated were involved in the TLE development. Besides, there was a significant correlation between hub DEIRGs and some of the specific immune cells.

**Conclusion:**

4 hub DEIRGs (Ptgs2, Jun, Icam1, Il6) were associated with the pathogenesis of TLE *via* regulation of immune cell functions, which provided a novel perspective for the understanding of TLE.

## Introduction

Epilepsy is a chronic neurological disease in which patients suffer from the recurrence of unprovoked seizures and neurocognitive deficits, impacting more than 70 million people’s life worldwide (Englot et al., 2020). Temporal lobe epilepsy (TLE) is a common type of intractable epilepsy. Previous reports have indicated significant changes in multiple cognitive domains, such as judgment, problem-solving, language, executive functions, and memory (Hermann et al., 2006; Keary et al., 2007; Allone et al., 2017). Although lots of genes and pathways involved in the etiology of TLE have been widely reported, the precise pathological mechanisms are still unclear. Besides, there are no effective therapeutic targets or precise biomarkers for the occurrence and development of TLE.

Accumulating evidence has revealed that neuroinflammatory, oxidative stress, and excitotoxicity contributed to posttraumatic epileptogenesis (Ambrogini et al., 2019; Eastman et al., 2020). Neuroinflammatory is one of the major biological processes in the development of brain pathology in epilepsy (Paudel et al., 2018; Rana and Musto, 2018). The inflammatory response to the insult induced a cascade of processes resulting in multiple pathological effects, including neurodegeneration, astrocyte dysfunction, damaged blood-brain barrier, long-term plastic alterations, and neuronal excitability. These lesions ultimately resulted in the occurrence and development of TLE (Suleymanova, 2021). Activation of neuroinflammation is commonly presented in epileptogenic brain areas and is implicated in animal models of epilepsy (Okuneva et al., 2016). Besides, IRGs in the molecular imaging and blood of neuroinflammation could provide diagnostic and predictive biomarkers for epilepsy (Vezzani et al., 2019). Furthermore, anti-inflammatory small molecules had the potential therapeutic effects of inhibiting brain inflammation in the treatment of epilepsy (Radu et al., 2017). The inflammatory responses induced the secretion of pro-inflammatory factors, which resulted in blood-brain barrier dysfunction as well as the involvement of resident immune cells in the inflammation pathway (Matin et al., 2015). Experimental models have demonstrated that neural injury and the onset of recurrent epilepsy are regulated by interactions between adaptive and innate immunity (Falsaperla et al., 2014; Vitaliti et al., 2014). Immunocyte infiltration takes part in the development of epilepsy primarily via exacerbating blood-brain barrier damage and activating brain inflammation (Wang et al., 2014). For example, the expression of the chemokines positively correlated with neutrophil infiltration in a rat seizure model (Johnson et al., 2011). Infiltration of T lymphocytes is also implicated in the pathology of epilepsy (Nakahara et al., 2010). Furthermore, PD-1 is an immune-inflammatory diagnostic biomarker for patients with intractable epilepsy (Tang and Wang, 2021). Based on these studies, an inflammatory- and immune-related therapeutic schedule could be effective in the prevention and treatment of TLE.

In the present study, we identified inflammation-related genes (IRGs) from the GSE88992 dataset using a combined approach of differentially expressed gene analysis and weighted gene coexpression network analysis. Subsequently, we identified the differential immunocyte types associated with TLE development. And the relationship between immune cell infiltrates and IRGs were evaluated.

## Materials and methods

### Collection of microarray data

We downloaded the gene expression profiles of the GSE88992 dataset (*Mus musculus*) with 9 control samples and 8 TLE samples from the Gene Expression Omnibus (GEO) database.^1^ The mouse model of TLE was established by intrahippocampal microinjection of kainate. We collected the IRGs list from the GeneCards database,^2^ the threshold for IRGs screening was set as follows: the relevance score > 5. GSE60772 dataset (*Mus musculus*) was used as an external dataset. The microarray data were normalized with the normalize between arrays function of the limma package.

### Screening of differentially expressed genes

We used the limma package of R software to identify the DEGs between the control and TLE groups. The screening criteria were p.adj < 0.05 and |log2 FC| > 1. The volcano and heatmap of DEGs were generated by the ggplot2 package and the Complex Heatmap package in R software, respectively.

### Weighted gene co-expression network analysis

We used the WGCNA R package to carry out the WGCNA analysis (Langfelder and Horvath, 2008). A total of 20,812 genes were identified from each sample of GSE88992 dataset. Firstly, the Pearson correlation coefficient between every two genes was calculated to construct the similarity matrix. In the present study, we selected the power value β = 6 (scale free *R*^2^ = 0.91) and mean connectivity = 10.54. Then, the similarity matrix was transformed into a topological overlap matrix (TOM). Next, hierarchical clustering was performed to identify modules, the minimum size cutoff value was 30 and the depth segmentation value was 3. The eigengene was calculated, the modules were clustered, and the similar modules were merged (module merge threshold = 0.25). Subsequently, the gene modules that were related to the clinical traits of TLE were identified. Finally, the potential hub genes were identified based on the criteria: gene significance (GS) > 0.8 and module significance (MS) > 0.8.

### Functional enrichment analysis of inflammation-related genes

The cluster Profiler package of R software was used to perform the KEGG and biological process (BP), cellular component (CC), and molecular function (MF) enrichment analyses of DEIRGs. *P* < 0.05 was considered significant enrichment (Yu et al., 2012).

### Identification of hub genes

First, the PPI network of IRGs was established using a STRING^3^ and Cytoscape (v 3.7.2) software. Then, we selected the top 8 genes by eight topological analysis algorithms in cytoHubba, namely, radiality, MNC, EPC, MCC, degree, closeness, betweenness, and bottleneck (Chin et al., 2014). The hub genes were identified using an R package “UpSet” (Lex et al., 2014).

### Gene set enrichment analysis

GSEA was carried out *via* GSEA version 4.1.0 software to further explore the potential biological functions of genes in the GSE88992 dataset (Tragante et al., 2017). False discovery rate (FDR) < 0.25 and p.adjust < 0.05 and were considered significant enrichment.

### Assessment of immunocyte infiltration

The gene expression profiles of the GSE88992 dataset were uploaded to CIBERSORT to evaluate immune cell infiltration of TLE samples and control samples (Newman et al., 2015). The ggplot2 package and the ComplexHeatmap package in R software were used to visualize the infiltration level of 22 types of immune cells between the control and TLE groups.

### Correlation analysis between the infiltrating immune cells and hub genes

The correlation analysis between the infiltrating immune cells and hub genes (Ptgs2, Jun, Icam1, and Il6) was carried out *via* Spearman, the results were visualized *via* the ggplot2 package of R software.

### The kainite model of temporal lobe epilepsy and hub genes validation

The male C57BL/6 mice (8 weeks old and 20–24 g) were obtained from the Experimental Animal Center of Chongqing. All animal experimental protocols were approved by the Animal Care Committee of Army Medical University. All animals were randomly divided into two groups, including control group (Con, *n* = 6) and kainite model group (TLE, *n* = 6). Mice were anesthetized with a combination of xylazine (8 mg/kg, i.p.) and ketamine (100 mg/kg, i.p.), and placed symmetrically in the stereotactic apparatus and then drilled into the skull. Then, freshly prepared kainite solution (20 mmol/L in saline) (Araki et al., 2020) was microinjected into the right side of the hippocampus by a microsyringe to induce TLE. The microsyringe was slowly withdrawn and the scalp was stitched. The Con of mice was given the same amount of saline. During the first 4 h after kainite injection, the severity of TLE was evaluated based on the Racine scoring system: 0, no reaction; 1, mild facial clonus, eye blinking, and/or stereotypic mounting; 3, myoclonic jerks in the forelimbs; 4, clonic convulsions in the forelimbs with rearing; and 5, generalized clonic convulsions and loss of balance (Khamse et al., 2015).

Total RNA of hippocampus tissues were extracted using TRIzol reagent (Ambion). 2 μg of purified RNA was reverse transcribed into cDNA using cDNA synthesis kit (Bio-Rad, USA). Then, qRT-PCR was carried out using SYBR Green Master Mix reagent (VAZYME) on the PCR System (Applied Biosystems, CA, USA). The mRNA expression levels of genes were normalized using the 2^–ΔΔ^*^Ct^* method. The sequences of primers used in this study were presented in Table 1.

**TABLE 1:** Sequences of primers used quantitative real-time PCR.

Gene	Primer	Sequence (5′–3′)	PCR products
Mus β-actin	Forward	CACGATGGAGGGGCCGGACTCATC	240 bp
	Reverse	TAAAGACCTCTATGCCAACACAGT	
Mus Ptgs2	Forward	GTGATGAGCAACTATTCCA	311 bp
	Reverse	GGTGAATGACTCAACAAAC	
Mus Jun	Forward	CGGATCAAGGCAGAGAGGAA	221 bp
	Reverse	GTTAGCATGAGTTGGCACCC	
Mus Icam1	Forward	AAGATGACCTGCAGACGGAA	231 bp
	Reverse	ATAAGAGGCTGCCATCACGA	
Mus Il6	Forward	GTTCTCTGGGAAATCGTG	254 bp
	Reverse	AGGACTCTGGCTTTGTCT	

### Hub gene-drug interaction

Drug-Gene Interaction Database has drug-gene interaction data from 30 disparate sources including PharmGKB, NCBI Entrez, clinical trial databases, Ensembl, DrugBank, ChEMBL, and literature in NCBI PubMed. The four core IRGs were selected as the potential targets and imported into the Drug-Gene Interaction Database (DGIdb)^4^ to search for existing inhibitors or agonists, then the FDA-approved drugs with agonist or antagonist effects were identified. Finally, the interaction network between the core IRGs and the potential drugs was constructed by Cytoscape software.

## Results

### Identification of differentially expressed genes between temporal lobe epilepsy and control groups

**FIGURE 1:**
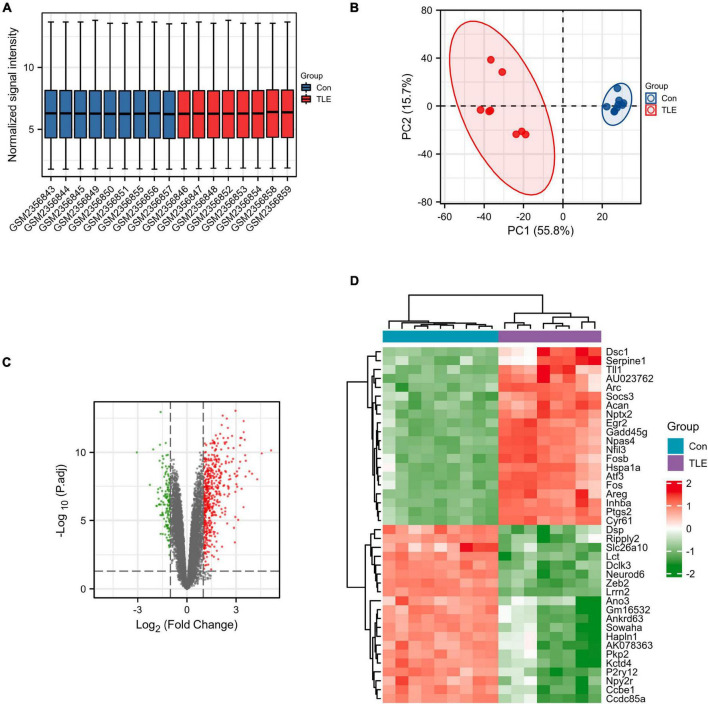
Identification of DEGs in TLE. **(A)** Normalized data from control and TLE groups. **(B)** Principal component analysis for GSE88992. **(C)** Volcano plot of the DEGs (green dots represent 139 down-regulated genes, red dots represent 474 up-regulated genes). **(D)** Heatmaps of the top 40 DEGs in the GSE88992 dataset. Red indicates up-regulated and green indicates down-regulated.

First, the data from the GSE88992 dataset were normalized with the normalize between arrays function of the limma package (Figure 1A). And the results of PCA analysis indicated a significant difference in gene expression between control samples and TLE samples (Figure 1B). Therefore, we performed DEGs analysis in the next step. As shown in Figure 1C and Supplementary Table 1, we identified a total of 613 DEGs between TLE and control groups, including 474 up-regulated genes (red dots) and 139 down-regulated genes (green dots). The mRNA expression level of the top 40 DEGs was visualized in Figure 1D.

### Temporal lobe epilepsy-related modules were identified by weighted gene co-expression network analysis

A total of 20,812 genes obtained from the gene expression profiles of the GSE88992 dataset were used to perform WGCNA. Then, hub modules were identified based on average dynamic tree clipping and hierarchical clustering (Supplementary Figures 1A,B). Two hub modules (blue and turquoise) were visualized in Supplementary Figure 1C and were most correlated with the TLE and selected for further investigation. As shown in Supplementary Figures 1D,E, we screened out the hub genes based on MM > 0.8 and GS > 0.8, and 2,312 and 2,651 key genes were identified in the blue and turquoise modules, respectively (Supplementary Table 2).

### Identification of core inflammation-related genes and functional enrichment analysis

**FIGURE 2:**
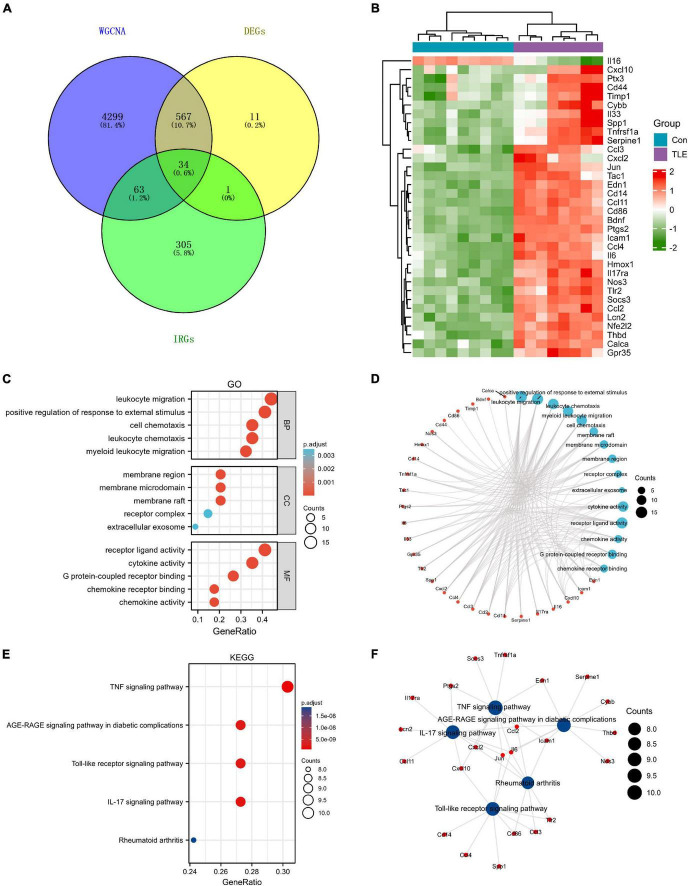
Identification of differentially expressed IRGs (DEIRGs) in TLE. **(A)** Venn diagram of the DEIRGs in DEGs, WGCNA, and IRGs. A total of 34 DEIRGs were identified. **(B)** Heatmaps of the 34 DEIRGs in the GSE88992 dataset. Red indicates up-regulated and green indicates down-regulated. The results showed that most of DEIRGs were all up-regulated in TLE group. **(C,D)** Biological process (BP), cellular component (CC), and molecular function (MF) enrichment analyses of DEIRGs. **(E,F)** KEGG enrichment analysis of DEIRGs.

A total of 403 IRGs were obtained from GeneCards database (Supplementary Table 3). We intersected the most clinically significant modules (blue and turquoise) with the DEGs and IRGs to further identify 34 core IRGs (Figure 2A). The mRNA expression level of the 34 core IRGs in the GSE88992 dataset was visualized in Figure 2B. Subsequently, we performed a functional enrichment analysis of 34 core IRGs to obtain a better understanding of the underlying BP associated with TLE. As shown in Figures 2C,D, the GO-BP enrichment analysis indicated that the core IRGs involved in leukocyte migration, positive regulation of response to external stimulus, leukocyte chemotaxis, myeloid leukocyte migration, and cell chemotaxis; for GO-CC, the core IRGs mainly enriched in membrane raft, membrane microdomain, membrane region, receptor complex, and extracellular exosome; for GO-MF, the core IRGs significantly enriched in cytokine activity, receptor ligand activity, chemokine activity, G protein-coupled receptor binding, and chemokine receptor binding (Table 2). As shown in Figures 2E,F, the KEGG enrichment analysis indicated that the core IRGs enriched in TNF signaling pathway, IL-17 signaling pathway, Toll-like receptor signaling pathway, AGE-RAGE signaling pathway in diabetic complications, and Rheumatoid arthritis (Table 2).

**TABLE 2:** Enrichment analysis of differentially expressed IRGs in kainite-induced epilepsy.

Ontology	ID	Description	GeneRatio	BgRatio	*P*-value	p.adjust	*q*-value
BP	GO:0050900	Leukocyte migration	15/34	340/23,210	3.25e−19	6.61e−16	2.83e−16
BP	GO:0032103	Positive regulation of response to external stimulus	14/34	312/23,210	5.14e−18	5.22e−15	2.23e−15
BP	GO:0030595	Leukocyte chemotaxis	12/34	211/23,210	1.07e−16	7.24e−14	3.10e−14
BP	GO:0097529	Myeloid leukocyte migration	11/34	207/23,210	5.20e−15	2.64e−12	1.13e−12
BP	GO:0060326	Cell Chemotaxis	12/34	299/23,210	7.14e−15	2.90e−12	1.24e−12
CC	GO:0045121	Membrane raft	7/34	364/2,3436	7.72e−07	2.34e−05	1.47e−05
CC	GO:0098857	Membrane microdomain	7/34	365/2,3436	7.86e−07	2.34e−05	1.47e−05
CC	GO:0098589	Membrane region	7/34	377/23,436	9.76e−07	2.34e−05	1.47e−05
CC	GO:0043235	Receptor complex	5/34	394/23,436	2.44e−04	0.003	0.002
CC	GO:0070062	Extracellular exosome	3/34	90/23,436	3.01e−04	0.003	0.002
MF	GO:0005125	Cytokine activity	12/34	215/22,710	1.74e−16	2.03e−14	1.02e−14
MF	GO:0048018	Receptor ligand activity	14/34	489/22,710	3.60e−15	2.10e−13	1.06e−13
MF	GO:0008009	Chemokine activity	6/34	44/22,710	4.79e−11	1.87e−09	9.41e−10
MF	GO:0001664	G protein-coupled receptor binding	9/34	293/22,710	3.46e−10	1.01e−08	5.10e−09
MF	GO:0042379	Chemokine receptor binding	6/34	67/22,710	6.61e−10	1.55e−08	7.79e−09
KEGG	mmu04668	TNF signaling pathway	10/33	113/8,910	5.21e−12	7.19e−10	4.22e−10
KEGG	mmu04657	IL-17 signaling pathway	9/33	91/8,910	2.55e−11	1.76e−09	1.03e−09
KEGG	mmu04620	Toll-like receptor signaling pathway	9/33	100/8,910	6.05e−11	2.29e−09	1.34e−09
KEGG	mmu04933	AGE-RAGE signaling pathway in diabetic complications	9/33	101/8,910	6.63e−11	2.29e−09	1.34e−09
KEGG	mmu05323	Rheumatoid arthritis	8/33	87/8,910	6.78e−10	1.87e−08	1.10e−08

### Identification and validation of hub genes

**FIGURE 3:**
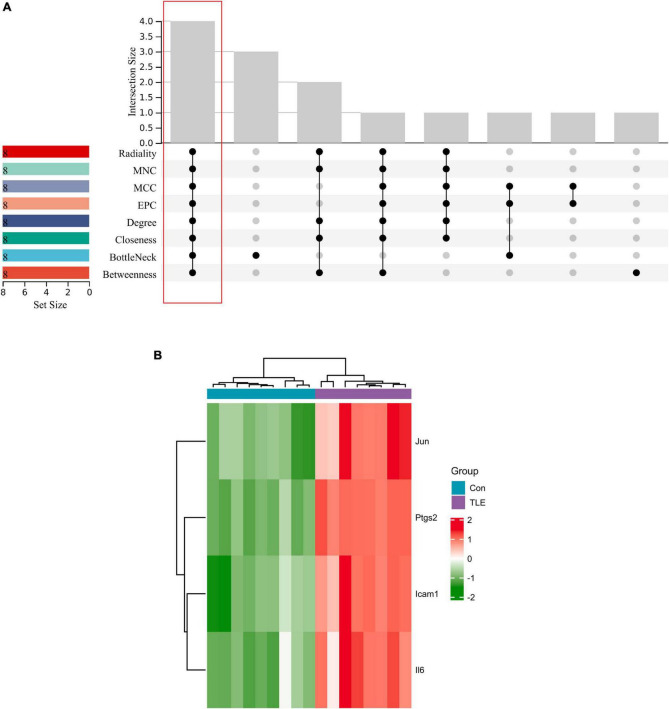
Identification of hub genes. **(A)** Eight algorithms were applied to identify hub genes based on the R package “UpSet.” Four IRGs (Ptgs2, Jun, Icam1, Il6) were identified as hub genes. **(B)** Heatmap of four hub genes (Ptgs2, Jun, Icam1, Il6) in the GSE88992 dataset. Red indicates up-regulated and green indicates down-regulated. The results showed that Ptgs2, Jun, Icam1, Il6 expression levels were all up-regulated in TLE group.

**FIGURE 4:**
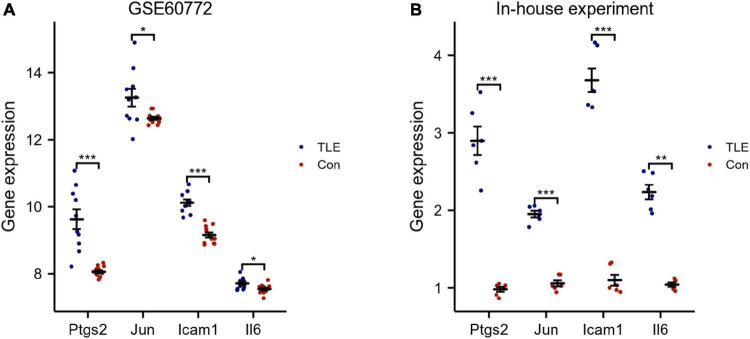
Validation of hub genes. **(A)** Validation of hub genes by an external dataset (GSE60772). **(B)** Verification of hub genes by an in-house experiment. The findings showed that Ptgs2, Jun, Icam1, Il6 expression levels were all increased in TLE group. Data were presented as mean ± standard deviation (SD). *n* = 3. *P < 0.05, **P < 0.01, ***P < 0.001.

The top 8 genes were selected by 8 topological analysis algorithms (radiality, MNC, EPC, MCC, degree, closeness, betweenness, and bottleneck) in cytoHubba. Then, we used the R package “UpSet” to identify four hub genes, including Ptgs2, Jun, Icam1, and Il6 (Figure 3A). We also drew the heatmap of four hub genes in the GSE88992 dataset to visualize the mRNA expression levels (Figure 3B). Besides, we used an external dataset (GSE60772) and established a model of TLE to verify the results. As presented in Supplementary Table 4, Con group showed no signs of seizure activity and behavior during the first 4 h after kainite injection. However, all mice (100%) in TLE group showed high scores of seizures (*p* < 0.001), indicating that the TLE model was established. As shown in Figures 4A,B, the mRNA expression levels of Ptgs2, Jun, Icam1, and Il6 in the control group were significantly lower than those in the TLE group (*p* < 0.05), the results were consistent with those of bioinformatics screening.

### Gene set enrichment analysis

**FIGURE 5:**
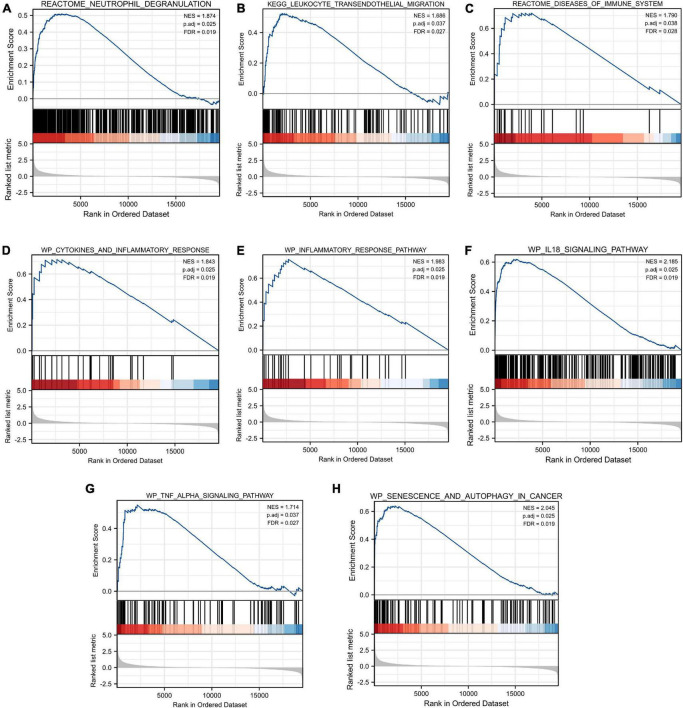
Gene Set Enrichment Analysis (GSEA). FDR < 0.25 and p.adjust < 0.05 were considered to be significant. **(A)** Neutrophil degranulation, **(B)** leukocyte transendothelial migration, **(C)** disease of the immune system, **(D)** cytokines and inflammatory response, **(E)** inflammatory response pathway, **(F)** IL18 signaling pathway, **(G)** TNF alpha signaling pathway, and **(H)** senescence and autophagy in cancer were involved in the pathological process of TLE. The GSEA results showed that immune- and inflammation-related pathways were significantly associated with TLE development.

As shown in Figure 5, our results revealed that most of the enriched gene sets were involved in immune- and inflammation-related pathways, including neutrophil degranulation, leukocyte transendothelial migration, disease of the immune system, cytokines and inflammatory response, inflammatory response pathway, IL18 signaling pathway, TNF alpha signaling pathway, and senescence and autophagy in cancer. These findings indicated immune response and inflammation may play a vital role in the pathological process of TLE.

### Immune infiltration analysis

**FIGURE 6:**
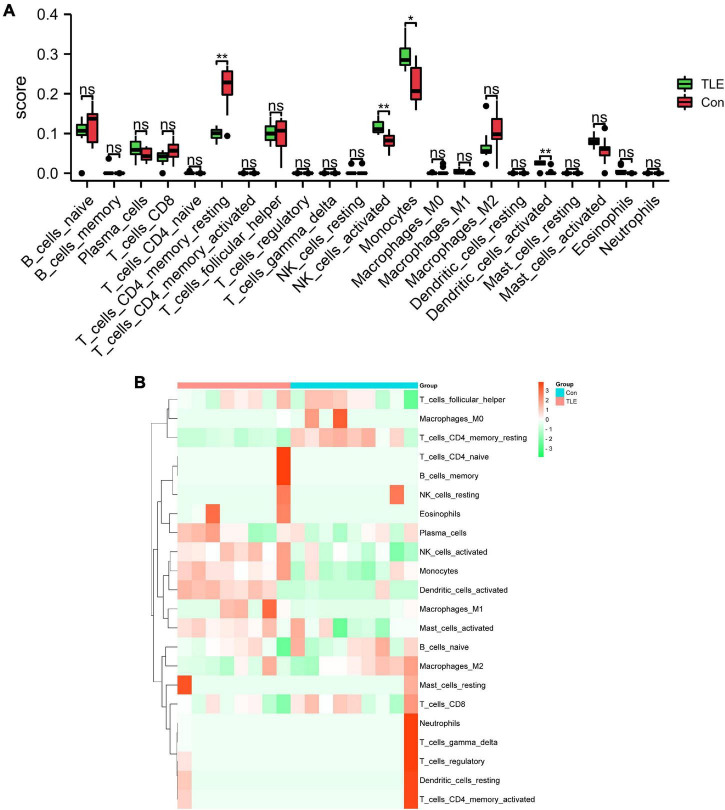
Analysis of immune cell infiltration landscape in TLE. **(A)** The histogram exhibits the differences in the score of the 22 types of immune cells between the control and TLE group in GSE88992 dataset. **(B)** Heatmap exhibited distribution of 22 types of immune cells between the control and TLE group in GSE88992 dataset. *P < 0.05, **P < 0.01.

In this study, we performed immune infiltration analysis to identify the immune cell types potentially involved in the pathology of TLE. As shown in Figure 6A, compared to those in the control group, the score of T cells CD4 memory resting and dendritic cells activated were relatively low, while the score of NK cells activated and monocytes were relatively high in the TLE group. The proportions of the 22 types of immunocyte cells were visualized in Figure 6B.

### Correlation analysis among Ptgs2, Jun, Icam1, Il6, and immune cells

**FIGURE 7:**
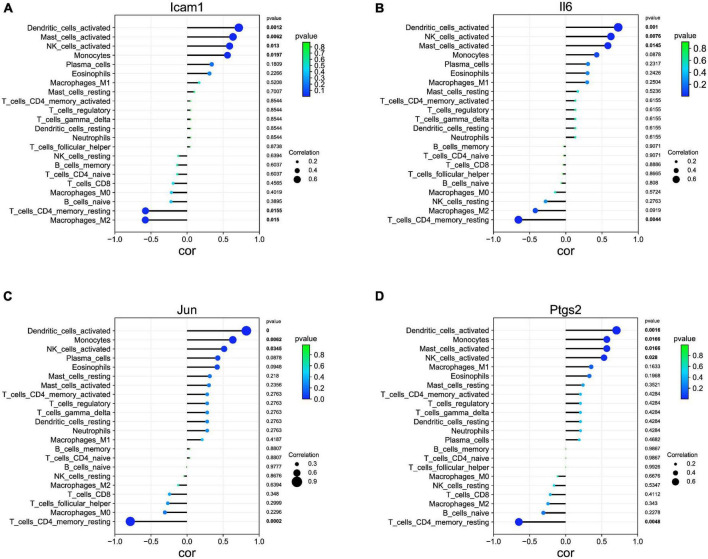
Association of hub genes with immune infiltrates. **(A)** Correlation between Icam1 and infiltrating immune cells. **(B)** Correlation between Il6 and infiltrating immune cells. **(C)** Correlation between Jun and infiltrating immune cells. **(D)** Correlation between Ptgs2 and infiltrating immune cells.

Our correlation analysis indicated that Icam1 was negatively with macrophages M2 (*r* = −0.578, *p* = 0.015) and T cells CD4 memory resting (*r* = −0.575, *p* = 0.015), and positively correlated with dendritic cells activated (*r* = 0.715, *p* = 0.001), mast cells activated (*r* = 0.634, *p* = 0.006), NK cells activated (*r* = 0.588, *p* = 0.013), and monocytes (*r* = 0.558, *p* = 0.019) (Figure 7A). Il6 was negatively with T cells CD4 memory resting (*r* = −0.654, *p* = 0.004), and positively correlated with Dendritic cells activated (*r* = 0.723, *p* = 0.001), NK cells activated (*r* = 0.622, *p* = 0.007), and Mast cells activated (*r* = 0.580, *p* = 0.014) (Figure 7B). Jun was negatively with T cells CD4 memory resting (*r* = −0.786, *p* = 0.0002), and positively correlated with Dendritic cells activated (*r* = 0.822, *p* = 0.0000), Monocytes (*r* = 0.634, *p* = 0.006), and NK cells activated (*r* = 0.514, *p* = 0.034) (Figure 7C). Ptgs2 was negatively with T cells CD4 memory resting (*r* = −0.649, *p* = 0.004), and positively correlated with Dendritic cells activated (*r* = 0.705, *p* = 0.001), Monocytes (*r* = 0.571, *p* = 0.016), Mast cells activated (*r* = 0.571, *p* = 0.016), and NK cells activated (*r* = 0.531, *p* = 0.028) (Figure 7D).

### Hub gene-drug interaction

**FIGURE 8:**
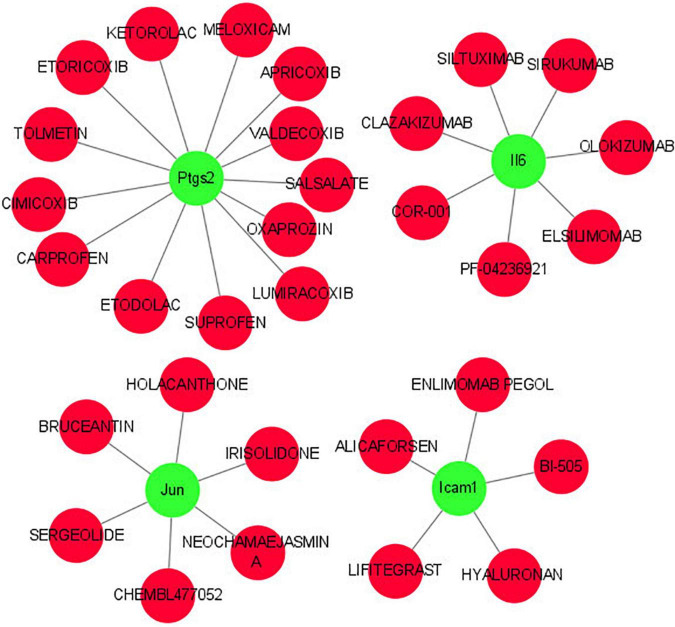
Interaction network between core IRGs and potential drugs. Green nodes represent the hub IRGs (Ptgs2, Jun, Icam1, Il6); red nodes represent the potential drugs for TLE.

As shown in Figure 8, we investigated the hub gene-drug interactions of the 31 potential drugs and 4 hub genes for possibly treating TLE. As shown in Table 3, the potential 31 candidate drugs were all approved by FDA. Most of these potential drugs might interact with the Ptgs2 (13/31). Promising drugs of these hub genes included ETORICOXIB, ALICAFORSEN, SILTUXIMAB, OLOKIZUMAB, etc.

**TABLE 3:** Candidate drugs targeted by the hub genes.

No.	Gene	Drug	Interaction types	Interaction score
1	Ptgs2	ETORICOXIB	Inhibitor	9.4
2	Ptgs2	CARPROFEN	Inhibitor	7.96
3	Ptgs2	VALDECOXIB	Inhibitor	5.06
4	Ptgs2	ETODOLAC	Inhibitor	3.13
5	Ptgs2	SUPROFEN	Inhibitor	2.89
6	Ptgs2	OXAPROZIN	Inhibitor	2.41
7	Ptgs2	APRICOXIB	Inhibitor	2.17
8	Ptgs2	SALSALATE	Inhibitor	2.17
9	Ptgs2	KETOROLAC	Inhibitor	2.17
10	Ptgs2	LUMIRACOXIB	Inhibitor	2.17
11	Ptgs2	TOLMETIN	Inhibitor	2.17
12	Ptgs2	CIMICOXIB	N/A	2.17
13	Ptgs2	MELOXICAM	Inhibitor	2.17
14	Jun	IRISOLIDONE	N/A	2.89
15	Jun	HOLACANTHONE	N/A	2.89
16	Jun	SERGEOLIDE	N/A	2.89
17	Jun	NEOCHAMAEJASMIN A	N/A	2.89
18	Jun	BRUCEANTIN	N/A	2.89
19	Jun	CHEMBL477052	N/A	2.89
20	Icam1	ALICAFORSEN	N/A	25.46
21	Icam1	BI-505	Antagonist	19.1
22	Icam1	ENLIMOMAB PEGOL	Antagonist	12.73
23	Icam1	LIFITEGRAST	N/A	8.49
24	Icam1	HYALURONAN	N/A	4.24
25	Il6	SILTUXIMAB	Inhibitor	10.18
26	Il6	OLOKIZUMAB	Inhibitor	10.18
27	Il6	CLAZAKIZUMAB	Inhibitor	7.64
28	Il6	COR-001	N/A	2.55
29	Il6	PF-04236921	Inhibitor	2.55
30	Il6	ELSILIMOMAB	Inhibitor	2.55
31	Il6	SIRUKUMAB	Inhibitor	2.55

## Discussion

Epilepsy is a brain disorder characterized by recurrent seizures. Imbalanced regulation of inflammation contributes to epilepsy development (Rana and Musto, 2018). Neuroinflammation is associated with an increased risk for epilepsy in related animal models and humans (Terrone et al., 2020). Increasing evidence indicated that systemic autoimmune disorders are the major pathogenesis of epilepsy (Steriade et al., 2021). Investigating the biological functions of immunity and inflammation in epileptogenesis will help in the identification of novel biomarkers for better screening of patients with epilepsy. Therefore, it is of great importance to further understand the role of IRGs and immune cell infiltration in epilepsy, and thus identify novel therapeutic targets. The present study aimed to systematically assess potential IRGs and investigated the correlations of immune cell infiltration in TLE.

In the present study, we screened 613 DEGs between TLE and the control group *via* bioinformatics methods. Then, we constructed a weighted co-expression network using WGCNA and identified hub modules for TLE, and four IRGs (Ptgs2, Jun, Icam1, Il6) were identified as hub genes. Besides, the proportions of T cells CD4 memory resting, dendritic cells activated, NK cells activated and monocytes were significant differences between TLE and control group, implying that immune response is involved in the TLE development. Furthermore, our results showed that these four IRGs were associated with immune cell infiltration in TLE, which could guide the selection of immunotherapy in the treatment of TLE.

An important result of this research was that the up-regulation of Ptgs2, Jun, Icam1, and Il6 was closely associated with the development of TLE. Prostaglandin-endoperoxide synthase 2 (Ptgs2) plays an important role in regulating inflammatory responses. It has been reported that Ptgs2 is dynamically regulated during the initiation and resolution of acute inflammation (Hellmann et al., 2015). Besides, down-regulation of peripheral PTGS2 in response to valproate treatment in epileptic (Rawat et al., 2020). Jun is a proto-oncogene, which encoded a variety of key regulatory proteins in the normal tissues. A previous study has indicated that Jun had the potential to serve as a prognostic marker for neuropathic pain (Yang et al., 2018). JUN was identified as a potential biomarker for the prognosis of brain low-grade gliomas (Xu et al., 2020). Besides, the c-JUN-medicated signaling pathway was an important BP involved in the pathogenesis of hippocampal neuronal damage in kainite-induced epilepsy (Lee et al., 2001). Intercellular adhesion molecule 1 (Icam1) is a cell surface glycoprotein that was reported for driving inflammatory responses in the onset of pathologic conditions. For example, increases in soluble Icam1 were associated with inflammatory responses, and some clinical studies used soluble Icam1 as a biomarker to classify patients with inflammatory diseases, as well as non-infectious systemic inflammatory responses syndrome (de Pablo et al., 2013). Icam1 was also reported to play an important role in regulating leukocyte trafficking and transendothelial migration (Kanters et al., 2008). Besides, expression of Icam1 was significantly up-regulated in the hippocampi with hippocampal sclerosis (Nakahara et al., 2010). Interleukin-6 (Il6) is a prototypical proinflammatory cytokine for maintaining homeostasis, and animal experiments showed the involvement of cytokines in epilepsy (Shen et al., 2020). The previous report has revealed that an increased level of IL6 was observed in cerebrospinal fluid and serum from patients with epilepsy (Peltola et al., 1998; Tao et al., 2020). Therefore, we speculated that these hub genes may play an important role in epilepsy.

Accumulating evidence has indicated that the activation of immune responses in the brain may be implicated in epilepsy (Granata et al., 2011). Peripheral immune cell invasion into the brain, along with cerebrovascular dysfunction and neuroinflammation, was implicated in the pathogenesis of epilepsy (Yamanaka et al., 2021). A previous study has reported that leukocytes infiltration in brain tissue is involved in the pathogenesis of TLE (Zattoni et al., 2011). As previously indicated, both microglial proliferation and monocytes recruitment resulted in significant microgliosis following epilepsy (Feng et al., 2019). Besides, infiltrating monocytes play an important role in the pathological process of epilepsy (Bosco et al., 2020). It has been reported that immunodeficient (depletion of T cells CD4) mice exhibited significant shortening of the latent stage before epilepsy onset, suggesting the protective effect of T cells in epileptogenesis (Deprez et al., 2011). Furthermore, compared with the healthy individuals, a significant increase of natural killer (NK) cells, while a significant decrease of CD4 T cells was observed in patients with TLE (Bauer et al., 2008). These results were in agreement with our findings. We also used CIBERSORT to comprehensively assess TLE immune cell infiltration to further investigate the role of immunocyte infiltration in TLE development. In this study, the score of T cells CD4 memory resting and dendritic cells activated were relatively low, while the score of NK cells activated and monocytes was relatively high in the TLE group. Moreover, the correlation analysis between Ptgs2, Jun, Icam1, Il6, and immune cells indicated that all these hub gene expressions were positively correlated with NK cells activated and negatively correlated with T cells CD4 memory resting. These findings further indicated the importance of immunocyte infiltration in the pathogenesis of TLE. We speculated that up-regulation of Ptgs2, Jun, Icam1, and Il6 contributes to NK cells activated infiltration and inhibits T cells CD4 memory resting infiltration, and subsequently results in the development of TLE. However, these results require additional *in vivo* or *in vitro* experiments to prove the complex interactions between hub genes and immunocyte infiltration.

## Conclusion

In conclusion, the present study combined WGCNA and DEG analyses to identify potential genes (Ptgs2, Jun, Icam1, and Il6) associated with specific immune cells involved in TLE development. Up-regulation of Ptgs2, Jun, Icam1, and Il6 contributes to TLE development *via* regulation of T cells CD4 and NK cells. Our findings provided a novel insight into the immunocyte infiltration pattern of TLE and its potential immune regulatory mechanisms.

## Data availability statement

The datasets presented in this study can be found in online repositories. The names of the repository/repositories and accession number(s) can be found below: https://www.ncbi.nlm.nih.gov/geo/, GSE88992 and GSE60772.

## Ethics statement

The animal study was reviewed and approved by the Animal Care Committee of Army Medical University.

## Author contributions

LW took part in drafting the manuscript. CD made substantial contributions to conception and design. RW and LC performed data analysis and interpretation of data. YW revised it critically for important intellectual content and gave final approval of the version to be published. All authors have read and agreed to the published version of the manuscript.

## Footnotes

1
https://www.ncbi.nlm.nih.gov/

2
https://www.genecards.org/

3
https://string-db.org

4
http://www.dgidb.org/

## Supplementary material

Supplementary Figure 1 Construction of WGCNA modules. **(A)** Dendrogram of all genes clustered based on a dissimilarity measure. **(B)** Heatmap of the eigengene adjacency. **(C)** Heatmap of the module-trait relationships. **(D)** Scatter plot for correlation analysis between gene significance and gene module membership in the blue module. **(E)** Scatter plot for correlation analysis between gene significance and gene module membership in the turquoise module.
