# Supplementary material for: Inflammation-related genes and immune infiltration landscape identified in kainite-induced temporal lobe epilepsy based on integrated bioinformatics analysis
Source: Front Neurosci. 2022 Oct 27;16:996368. doi: 10.3389/fnins.2022.996368 (PMC9648357; doi:10.3389/fnins.2022.996368)
Supplement: Supplementary file 4 [file Table_4.DOC]

Table S4 During the first 4 hours after kainite injection, the numbers and rates of mice with status epilepticus.

| Groups | Number of mice with status epilepticus | Epileptic seizure score | Rate (%) |
| --- | --- | --- | --- |
| Con | 0/6 | 0 | 0 |
| TLE | 6/6 | 4.67 ± 0.52*** | 100 |

****P* < 0.001.
