# Supplementary figures and images for: Inflammation-related genes and immune infiltration landscape identified in kainite-induced temporal lobe epilepsy based on integrated bioinformatics analysis
Source: Front Neurosci. 2022 Oct 27;16:996368. doi: 10.3389/fnins.2022.996368 (PMC9648357; doi:10.3389/fnins.2022.996368)

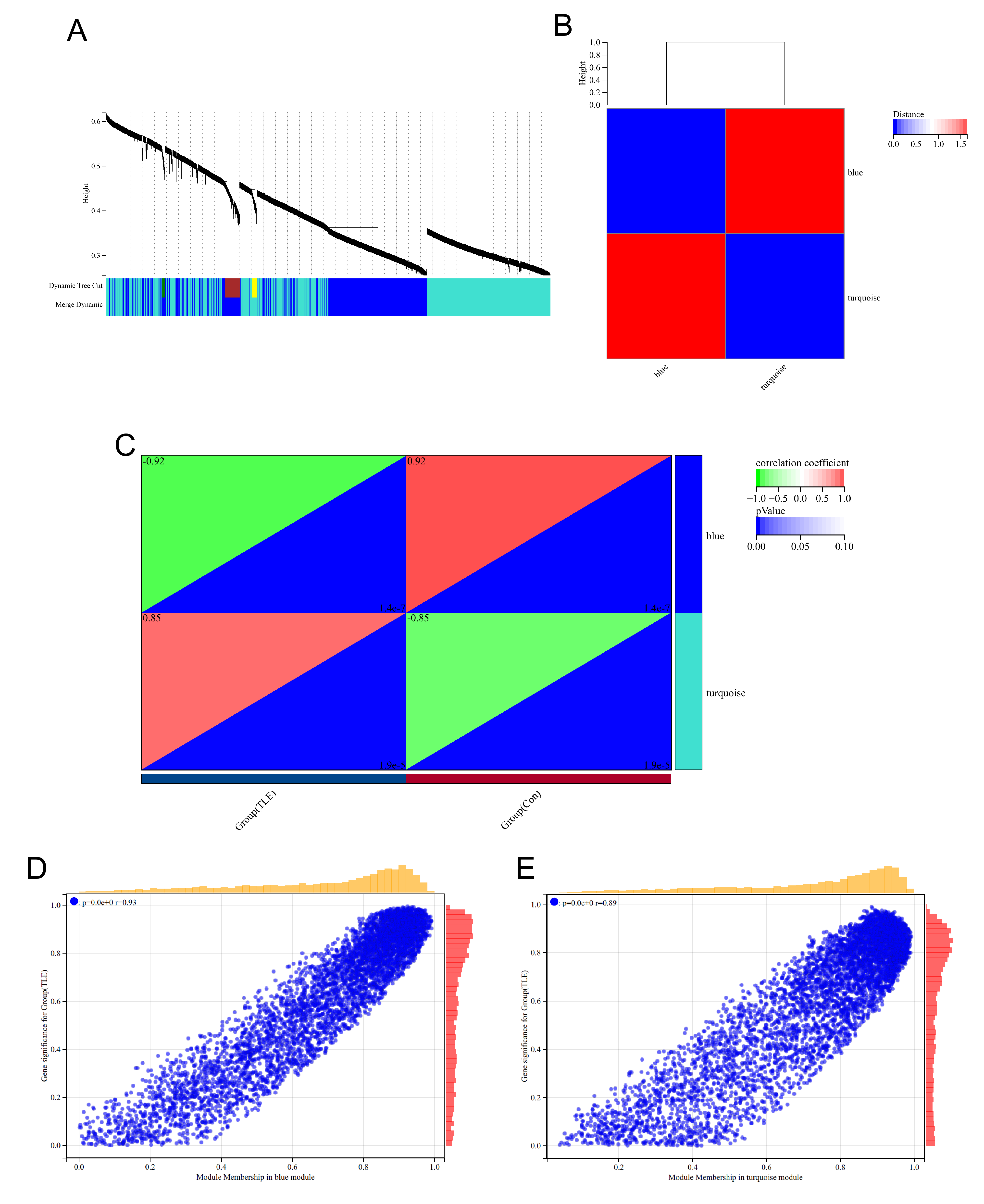

Supplement: Supplementary Figure 1 — Construction of WGCNA modules. (A) Dendrogram of all genes clustered based on a dissimilarity measure. (B) Heatmap of the eigengene adjacency. (C) Heatmap of the module-trait relationships. (D) Scatter plot for correlation analysis between gene significance and gene module membership in the blue module. (E) Scatter plot for correlation analysis between gene significance and gene module membership in the turquoise module. [file Image_1.tiff]
